# Editorial: Immunological Role of the Maternal Microbiome in Pregnancy

**DOI:** 10.3389/fimmu.2021.703009

**Published:** 2021-06-21

**Authors:** Nicoletta Di Simone, Eytan Robert Barnea, Martin Mueller

**Affiliations:** ^1^ Department of Biomedical Sciences, Humanitas University, Milan, Italy; ^2^ IRCCS Humanitas Research Hospital, Milan, Italy; ^3^ S.I.E.P. The Society for the investigation of Early Pregnancy, New York, United States; ^4^ Department of Obstetrics and Gynecology, University Hospital Bern, University of Bern, Bern, Switzerland; ^5^ Department of Pediatrics, Maastricht University Medical Centre (MUMC), Maastricht, Netherlands

**Keywords:** microbiota, immunity, pregnancy, implantation, preterm (birth), preterm/full term infants

In this Research Topic of Frontiers in Mucosal Immunity we have focused on the immunological role of the maternal microbiome in pregnancy. We have invited leading researchers in the medical field to contribute articles that summarize the advancements in this broad and complex topic. The understanding of microbiota and its function has been steadily expanding and recent advancements in molecular biology and the Human Microbiome Project have allowed us to take a new biological look on the microbiota and the host interactions. In case of pregnancy, the host has to be separated in two compartments namely the mother and the fetus. Furthermore, pregnancy itself induces a number of changes, which include immunological, hormonal, and metabolic changes - necessary for the maternal adaptation process and fetal development. Undoubtedly, the maternal microbiome influences the course of pregnancy and fetal development beyond plain colonization. Therefore in pregnancy, we have to consider the potential implications of microbiota or it`s changes on both compartments (the maternal and fetal) and importantly visa-versa. We have therefore selected 10 review and original articles covering the important aspect of the field.

In this Research Topic we have selected two contributions reviewing the global aspects of maternal microbiota and pregnancy interaction (Di Simone et al.; Al-Nasiry et al.). The article written by Di Simone et al. summarizes the abnormal changes of gastrointestinal, vaginal, and endometrial microbiota in pregnancy and potential morbidities throughout the pregnancy. An example is the association between abnormally increased intestinal permeability and uterine innate immunity affecting obstetrical outcomes. Al-Nasiry et al. summarize the composition of reproductive tract microbiome in relation to pregnancy and focuses on host-microbiota immune interactions. Therefore, the readers will understand how microbiota changes impact clinical outcomes such as infertility, recurrent miscarriage, and placental syndromes. We have selected additional review articles addressing the implications of microbiota and pregnancy in more detail. The article written by Bardos et al. evaluates the changes specifically in early pregnancy such as the relationship between the microbiota and endometrium/embryo development. The potential implication of uterine microbiota on immunological responses and resulting placental syndromes such as preeclampsia or intrauterine growth restriction are discussed. Another example is the complex interaction between microbiota and uterine immune cells, which are the topic of the article written by Agostinis et al. The article discusses the role of microbiota in the control of uterine cells character and function in pregnancy and beyond. Beyond expected and classical approach to review the complex topic of maternal microbiome in pregnancy, we have selected three additional review articles to summarize evidence pregnancy related morbidities and treatment approaches. The potential of fecal microbiota transplantation is reviewed by Quaranta et al. and the implications in the postpartum period such as neonatal outcomes is reviewed by Tirone et al. Finally, the immunological role of uterine microbiota in postpartum hemorrhage is reviewed by Escobar et al. Together, we have selected seven review articles covering the topic of the maternal microbiome in pregnancy and beyond. To further dissect the complexity of maternal microbiome in pregnancy we have selected additional three original articles.

The selected original articles cover different aspects of immune responses in pregnancy. Using germfree mice, Faas et al. show different immunological adaptations to pregnancy modulated by microbiota. For example, they detected the increase in Treg and tendency to an increase in Th2 cells in conventional pregnant mice only. Another article by Cui et al. focuses on the role of ROP16_I_, a rhoptry protein of Toxoplasma gondii, and macrophage polarization. The findings suggest that ROP16_I_ might be a protective factor and involved in the M1–Th1 biased pathological process in the early phase of gestation. Finally, Mohr et al. provide a prospective Case-Control study investigating inflammation in pregnant women with periodontitis and preterm prelabor rupture of membranes (PPROM). They concluded that systemic inflammation, initiated and triggered by periodontal disease, might play a role in developing preterm prelabor rupture of membranes.

Together, we have selected 10 review and original articles dissecting the various aspects of maternal microbiome in pregnancy. Although the selected articles provide an overview in this complex topic, we have placed particular emphasis on the immunological changes and potential implications – having both the mother and the fetus in mind.

## Author Contributions

All authors contributed equally. All authors contributed to the article and approved the submitted version.

## Conflict of Interest

The authors declare that the research was conducted in the absence of any commercial or financial relationships that could be construed as a potential conflict of interest.

