# Corrigendum: Editorial: Immunological role of the maternal microbiome in pregnancy

**DOI:** 10.3389/fimmu.2022.1109534

**Published:** 2022-12-14

**Authors:** Nicoletta Di Simone, Eytan R. Barnea, Martin Mueller

**Affiliations:** ^1^ Department of Biomedical Sciences, Humanitas University, Milan, Italy; ^2^ IRCCS Humanitas Research Hospital, Milan, Italy; ^3^ S.I.E.P. The Society for the investigation of Early Pregnancy, New York, United States; ^4^ Department of Obstetrics and Gynecology, University Hospital Bern, University of Bern, Bern, Switzerland; ^5^ Department of Pediatrics, Maastricht University Medical Centre (MUMC), Maastricht, Netherlands

**Keywords:** microbiota, immunity, pregnancy, implantation, preterm (birth), preterm/full term infants

In the published article, an author name was incorrectly written as Nicoletta DiSimone. The correct spelling is Nicoletta Di Simone.

The authors apologize for this error and state that this does not change the scientific conclusions of the article in any way. The original article has been updated.

